# Operationalizing a Framework for Appraising Seriousness to Guide Decision‐Making in Prenatal Care

**DOI:** 10.1002/jgc4.70318

**Published:** 2026-11-13

**Authors:** Erika Kleiderman, Anne‐Marie Laberge, Ainsley J. Newson, Lucinda Freeman, Vardit Ravitsky, Felicity Boardman

**Affiliations:** ^1^ Department of Social and Preventive Medicine, School of Public Health University of Montreal Montreal Canada; ^2^ Medical Genetics Division, Department of Pediatrics Centre Hospitalier Universitaire Sainte‐Justine and Université de Montréal Montreal Canada; ^3^ Faculty of Medicine and Health, Sydney School of Public Health, Sydney Health Ethics The University of Sydney Sydney NSW Australia; ^4^ Graduate School of Health University of Technology Sydney Sydney NSW Australia; ^5^ The Hastings Center Garrison New York USA; ^6^ Warwick Applied Health Directorate, Warwick Medical School University of Warwick Coventry UK

**Keywords:** assisted reproduction, prenatal care, prenatal decision‐making, reproductive autonomy, serious, severity

## Abstract

The seriousness of a genetic condition plays an important role in shaping expectant parents' understanding of results and options following prenatal testing. Yet it is well recognized that the decisions that follow such testing can be fraught. One key concern is that prenatal care professionals can emphasize biomedical or clinical interpretations of seriousness at the expense of more contextual elements as they relate to the circumstances and values of expectant parents. In this article, we draw on three exemplar clinical case studies to demonstrate how a framework for considering seriousness can be employed to expand the breadth and depth of conversations between prenatal care professionals and expectant parents, encourage shared meaning‐making around seriousness, and support expectant parents to navigate prenatal decision‐making in a way that aligns with their preferences, values, and circumstances. Our framework provides an underpinning structure to the conversations and conceptual spaces in which parental perceptions of seriousness are contextualized and animated. This, in turn, can lead to more effective counseling and improved decision‐making based on relevant and contextualized information.

## Abstract (highlights)

**What Is Known About This Topic:**

- Seriousness, an inherently subjective, multi‐faceted, and elusive concept, plays a key role in shaping expectant parents' prenatal decision‐making.
- A reliance and prioritization of biological or clinical factors pervade discussions and perceptions of seriousness.

**What Does This Article Add to the Topic:**

- Application of a novel framework that encourages shared meaning‐making between prenatal care professionals and expectant parents in how they understand and relate to seriousness.
- A focus on contextual elements and lived experience in appraisals of seriousness to more fully capture what is most relevant and useful to expectant parents.

## 1 Introduction

In prenatal testing, seriousness plays a key role in shaping expectant parents' understanding of both their particular results and the relevant condition more generally, as well as the reproductive options available to them (Bayefsky and Berkman 2022; Dive et al. 2023; Hewison et al. 2007; Ong et al. 2018; Plantinga et al. 2016; Sullivan et al. 2019). The medical paradigm often privileges biological or clinical facts over other forms of knowledge (such as patient‐reported quality of life) when seeking to determine a condition's seriousness (Boardman and Clark 2022; Savell and Karpin 2008; Kleiderman et al. 2019). For prenatal decision‐making, this prioritization of clinical information over contextual elements has a range of impacts, particularly with regard to women's reproductive autonomy and ability to make informed decisions (Savell and Karpin 2008).

It is widely recognized that support for patients faced with prenatal and reproductive decision‐making can be improved (Ouellette 2015; Swainson et al. 2024; Rutter et al. 2025). One major challenge is the complexity and volume of information. The rapid expansion of prenatal genetic testing options (e.g., non‐invasive prenatal testing,^1^ carrier screening, whole genome sequencing) has made it increasingly difficult for both expectant parents and prenatal care providers to navigate the information, especially as many tests can yield results of uncertain or unclear clinical significance or with limited ability to accurately predict the related future impact (Allyse and Chandrasekharan 2015). This is all the more relevant in the context of the global trend towards mainstreaming of genetic testing (Drysdale et al. 2024). A second challenge is that prenatal decisions are often time‐sensitive. This constriction of decision‐making timeframes can heighten emotional stress and limit the time available for comprehensive counseling and reflection, sometimes creating a perceived pressure to select for or against certain traits (Ouellette 2015; Ormond et al. 2009; Vanstone et al. 2018; Kater‐Kuipers et al. 2018). Moreover, access to genetic counseling remains limited due to a global shortage of trained professionals. As a result, non‐specialist healthcare professionals (e.g., general practitioners, obstetricians, nurses, and midwives) and, in some countries, patient organizations and/or charities are frequently called on to guide patients through complex decisions (Allyse et al. 2015; Bayefsky et al. 2016; Hoskovec et al. 2018). Such professionals and other stakeholders do not always have the necessary expertise and training to do this important task (Allyse and Chandrasekharan 2015). This status quo, and the evolving landscape of treatment options, underscore the need for ongoing education, training, and the development of tools to support healthcare professionals and other relevant bodies to engage in these sensitive and complex conversations effectively (Bayefsky et al. 2016; Borry et al. 2018; Chokoshvili et al. 2017). A further challenge is that variability in expectant parents' understanding and health literacy further complicates decision‐making processes, as individuals will bring diverse levels of comprehension, cultural backgrounds, or personal values that will shape how they perceive seriousness, assess probabilities, and make reproductive choices (Kibel and Vanstone 2017; Tsuge 2016).

Many have argued that clinical services that involve discussion about prenatal testing and reproductive choices should be reframed around a more balanced and patient‐centered approach that captures what is most relevant to the expectant parents (i.e., those who are considering becoming pregnant or those who are currently pregnant) (Ouellette 2015; Swainson et al. 2024; Rutter et al. 2025; Kleiderman et al. 2025; Taylor‐Sands et al. 2024; Sheets et al. 2011; Farrell and Allyse 2018). Swainson et al. further posit that “exploring a person's perception of the severity of a condition can provide a deeper understanding of how they are defining and understanding condition severity in the context of their lived experience, personal values and situation” (7) (Swainson et al. 2024). Therefore, in clinical care, considerations around the seriousness of a congenital or genetic condition should focus on the perspective of the expectant parents and what is “serious”^2^ to them. This framing is especially relevant when discussing the results of prenatal testing. To address these challenges, we have published a framework for considering seriousness in the context of prenatal and reproductive decision‐making (Kleiderman et al. 2025).

In this article, we demonstrate how the core dimensions of our framework can be operationalized in clinical care, to both better support conversations between prenatal care professionals and expectant parents, and to inform thinking around seriousness in the context of prenatal decision‐making. Although our framework is comprehensive, there remains a need for more detailed practical guidance, and this article directly assesses that gap. We consider these aspects using three clinical case studies: a couple who receive information about a prenatal diagnosis of Down syndrome; a couple who receive information about a prenatal diagnosis of spinal muscular atrophy; and a couple who are informed about a variant of uncertain significance. We contend that use of our framework can support all prenatal care professionals by offering a structured approach to discussing seriousness with expectant parents, within a wider social and cultural context, based on test results and the options that follow. In doing so, the framework promotes a more nuanced and inclusive understanding of genetic conditions and their seriousness. As a result, our framework is intended to be a tool for prenatal care professionals to engage in deliberative, patient‐centered dialogue and to maximize opportunities for expectant parents to make reproductive decisions that better align with their preferences, values, circumstances, and capacity to absorb and reflect on their results (White et al. 2021). Such flexibility is essential to delivering high quality, relevant support for expectant parents in contemporary genetic counseling, where decisions often intersect with complex social and ethical considerations, and where uniform definitions risk oversimplifying deeply personal and context‐dependent judgments (Ouellette 2015; Swainson et al. 2024; Taylor‐Sands et al. 2024).

## 2 Framing Conversations that Invoke Seriousness in Prenatal Care

### 2.1 Prenatal Decision‐Making

As noted above, prenatal diagnosis and decision‐making is often fraught with uncertainty, time pressure (where termination is a relevant consideration), an abundance of information (both complete and incomplete), and emotion as expectant parents navigate reproductive options that often require reflection on the seriousness of the diagnosed condition. Lived experience (experiential knowledge) and perceptions of seriousness have been shown to influence the acceptability of different reproductive options and what decisions expectant parents ultimately make (Dive et al. 2023; Swainson et al. 2024; Montgomery and Thayer 2020).

When working with expectant parents, it is important to ensure that what is being discussed is manageable, up‐to‐date, and captures the full spectrum of possibilities, especially if they do not have any prior experience (e.g., family history) or knowledge of the condition (Ouellette 2015; Swainson et al. 2024; Taylor‐Sands et al. 2024; Meredith et al. 2024; Gori et al. 2023). This includes providing an accurate and balanced representation of the condition, given that expectant parents often focus on the worst‐case scenario to drive their decision‐making, and particularly where a condition has a wide spectrum of presentations and/or where clinical information is ambiguous or uncertain (Swainson et al. 2024; Scruggs et al. 2024). Genetic counselors or clinical geneticists have experience, are knowledgeable, and are well equipped to counsel and provide expectant parents with relevant information on the condition being screened or tested for (Laforce et al. 2025). Yet, in practice, the limited availability of genetic counselors often necessitates that these discussions be undertaken by non‐genetics healthcare professionals, such as general practitioners, nurses, obstetricians, and midwives, who may lack the specialized knowledge or training required to comprehensively counsel and support expectant parents in interpreting and making informed decisions post‐result (Farrell and Allyse 2018; Long and Goldblatt 2014; Skirton et al. 2014). For both genetics professionals and non‐genetics healthcare professionals, our framework offers a practical structure for conversations with expectant parents regarding their conceptions of seriousness and how these relate to reproductive options. As we explore below, our framework is not a substitute for comprehensive and appropriate training, but can function as a structured prompt to ensure that both essential and relevant information is conveyed, and as a placeholder for spaces in which individuals can explore meanings, personal values, circumstances, and preferences.

Clinical decision‐making in reproduction should be oriented to support conversations or dialogue between expectant parents and their prenatal care professionals (Kleiderman et al. 2025; Gottfreðsdóttir and Árnason 2011; Gokcen et al. 2016). In so doing, it is meant to be an iterative and dynamic process (Montgomery and Thayer 2020). It is expected that decisions and perceptions of seriousness will vary from one individual to another, from couple to couple, and even for a given couple in different circumstances and timeframes, as demonstrated in the case studies below (Farrell and Allyse 2018). Expectant parents will also have different views on their expectations and aspirations for parenthood, which calls for a flexible approach to conceptualizing seriousness (Taylor‐Sands and Bowman‐Smart 2022).

### 2.2 A Framework for Considering Seriousness

Our framework comprises a set of four core dimensions and four procedural elements (see Figure 1). It serves as a conceptual tool to add breadth to discussions and support decision‐making that invokes seriousness (Kleiderman et al. 2025). The dimensions and elements map the contours of seriousness and foster a common understanding of its features to facilitate informed decision‐making (Kleiderman et al. 2025). The core dimensions—medical factors, access/availability of treatment, access/availability of support and resources, and individual and familial lived experiences—reflect more theoretical considerations related to seriousness or the factors to consider, whereas the procedural elements—patient/family‐centered impacts, shared decision‐making, assessment of training of healthcare professionals, and procedural fairness in assessing seriousness—reflect how the concept could be implemented in various contexts (Kleiderman et al. 2025). Table 1 provides a description and overview of the considerations related to each core dimension and procedural element.

**FIGURE 1:**
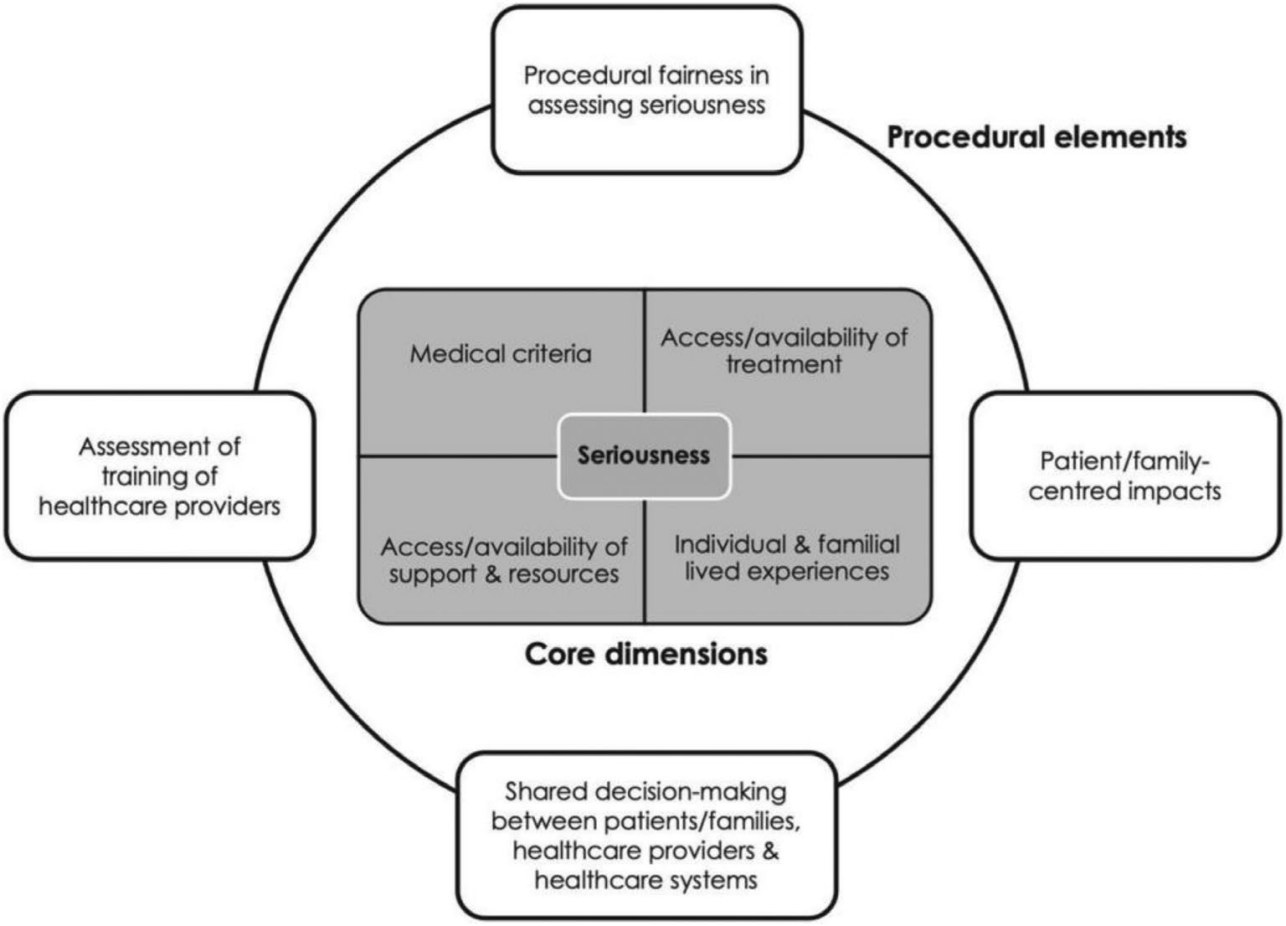
Core dimensions and procedural elements of seriousness. This figure is based on the following source: Kleiderman et al. (2025). Licensed under CC BY 4.0 (https://creativecommons.org/licenses/by/4.0/). No changes were made.

**TABLE 1:** Description and considerations of the core dimensions and procedural elements of seriousness.

**Core dimensions: Description/Considerations**	
Medical factors	Objective medical aspects or clinical manifestations, for example, morbidity, impact on life expectancy, diagnosis, type of disability (cognitive, physical, sensory), penetrance, expressivity, phenotypic range, age of onset
Access/availability of treatment	Availability and accessibility of treatment to mediate symptoms, including the goals of treatment (curative, prophylactic, supportive, experimental), burden of undergoing treatment (physical, interpersonal, financial), availability of resources, implications for distributive justice (out‐of‐pocket costs & prioritization of treatments)
Access/availability of support & resources	Types of mechanisms or support (counseling, social support, resources, familial support, infrastructure) built‐in or integrated into the healthcare system Include factors such as resource availability (social capital, financial stability), financial (socio‐economic status, cost of living with condition, impact on household productivity), impact of local context (differences between countries)
Individual & familial lived experiences	Testimony on lived experiences of people and families living with or caring for someone with the condition Include factors such as personal/identity (values, preferences, perceived quality of life, community), psycho‐social aspects (burden, ability to plan, impact on daily functioning), socio‐cultural (ethnicity, culture, language, religion/spirituality, societal perceptions, historical/legal/political contexts), built & physical environment (accessibility, geographic location)
**Procedural elements: Description/Considerations**	
Patient/family‐centered impacts	Impact on family & community (not limited to the individual, considerations around close environment & capacity to deal with changes) Influence of various factors (experiential factors, values & preferences; personal identification with the condition; socio‐cultural context)
Shared decision‐making between patients/families, healthcare professionals & healthcare systems	Capacity for listening & shared meaning‐making Foster dialogue about the impact of the decision on the expectant couple, future child & family Manageable & understandable information (sufficient time for discussion, openness in how information is presented – neutrality wherever possible, balanced examples & a non‐directive approach)
Assessment of training of healthcare professionals	Importance of appropriate & adequate training (ensuring healthcare professionals can effectively engage with patients) Limited availability of adequate counseling (scalability issues due to lack of genetic counselors) Recognize & mitigate potential biases or public value judgments
Procedural fairness in assessing seriousness	Transparency in decision‐making (clear motivations & objectives, evidence‐based, setting thresholds on permitted uses) Consideration of socio‐political contexts specific to a country or society Importance of clear language in laws & policies Recognize tension in categorizing a genetic condition as serious (individual vs. population perspectives)

*Note:* Adapted from Kleiderman et al. (2025).

The core dimensions and procedural elements are intended to be complementary, interrelated, and applied in a proportionate manner depending on the context. For example, the core dimensions would be more heavily weighted in prenatal care where the contextual aspects of a person's life and their lived experience directly influence how seriousness is conceptualized and understood to inform subsequent decision‐making. Contextual factors play a particularly critical role in prenatal diagnosis and decision‐making given that decisions involved are inherently personal, ethically complex, and often value‐laden and emotionally charged (Vanstone et al. 2018; Donley et al. 2012; Walser et al. 2015).

The framework enables prenatal care professionals to explore the core dimensions of seriousness with expectant parents, collaboratively assessing what matters most to them and why. The core dimensions can be explored in any order, based on the importance to the expectant parents. In this way, it serves as a tool to trigger guided reflections on the potential seriousness of a genetic condition or diagnosis, in ways that are attentive to its complexity. The framework's procedural elements complement and support these guided reflections by highlighting the considerations that are essential in order to make these reflections productive—leading to tailored genetic counseling that can best inform decision‐making throughout the pregnancy (Bayefsky and Berkman 2022; Swainson et al. 2024; Taylor‐Sands and Bowman‐Smart 2022). This acknowledges that expectant parents may require varying levels of information at different time points, and that their perceptions of seriousness may evolve as the pregnancy progresses.

Through its core dimensions and procedural elements, the framework does not aim to define seriousness, but to facilitate a collaborative exploration and operationalization of the concept in prenatal and reproductive decision‐making. Essentially, the framework provides an underpinning structure to the conversations between prenatal care professionals and expectant parents, in which parental perceptions of seriousness are contextualized and animated (Kleiderman et al. 2025). This encourages a fuller understanding of seriousness, even where expectant parents may not agree (with each other—if they are a couple—or with the prenatal care professional) on what is “serious” and what is not. For example, there can be agreement between expectant parents on what dimensions to consider when appraising seriousness and ways of thinking about the concept, although the weight given to each dimension may vary.

In our view, the framework is applicable in all scenarios of prenatal decision‐making and across the full spectrum of clinical severity—including conditions deemed extremely severe based on clinical presentation, such as early‐onset, life‐limiting conditions or those involving significant functional impairment (e.g., Tay–Sachs disease). The difference in such scenarios is the focus of the discussions, and which core dimensions are explored with expectant parents based on their circumstances and information needs, as all core dimensions may not apply. In these instances, the framework provides a helpful structure for prenatal care professionals to frame their conversations in a way that is responsive to the information needs of expectant parents. As noted earlier, this can be particularly beneficial when prenatal care professionals do not have the specialized knowledge or training to engage in depth conversations or dialogue with expectant parents on such topics.

In so doing, the framework supports greater integration of lived experience and the perspectives of those directly involved in and impacted by prenatal decision‐making. For the purpose of the framework, individual and familial lived experience refers to direct experiences and knowledge gained from personally living with a certain condition or from caring for someone with that condition. It is not intended to diminish or collapse these distinct experiences into a single category but to reflect that these personal factors can shape an individual's understanding and perception of the world. This patient‐centered approach builds on standard clinical practice by providing a way of structuring and approaching discussions around perceived seriousness and how expectant parents are contextualizing the information within their own lived experience and circumstances. For example, expectant parents' perceptions of health and disease are shaped by their embodied experiences, informing how they evaluate particular health states as either tolerable or intolerable within the context of their own lived realities. Adopting a more contextual approach may also improve inclusion of families with a wide range of social, cultural, or religious beliefs (Kleiderman et al. 2022). Therefore, the framework supports a flexible approach and a shift towards shared decision‐making or reproductive deliberation that highlights patient engagement through dialogue and information that is responsive to their needs (Barry and Edgman‐Levitan 2012; Warton et al. 2023).

## 3 Three Case Studies

We will now illustrate the application of our framework for considering seriousness through three hypothetical case studies. These case studies were developed by the co‐authors. Although they are hypothetical, they are consistent with our professional and personal experience. Case #1 (see Box 1) involves a couple receiving a prenatal diagnosis result indicating their fetus has Down syndrome (a chromosomal aneuploidy). Case #2 (see Box 2) involves a couple receiving a result indicating that their fetus will develop spinal muscular atrophy (a monogenic neuromuscular condition). Case #3 (see Box 3) involves a couple being informed that a variant of uncertain significance has been identified in their fetus. These cases illustrate how the framework can apply to conversations related to conditions with distinctions in terms of lifespan, treatability, and predictability of outcomes. The variability and subjectivity reflected in these scenarios illustrate the framework's capacity to accommodate diverse perspectives—not only regarding the concept of a condition's seriousness, but also in a broader, holistic sense. This multi‐dimensional approach ensures that appraisals of seriousness are not confined to biomedical factors but reflect the lived experiences and contextual realities of individuals and families.

A key challenge when providing counseling to expectant parents lies in creating a space for value‐based reflection without unduly influencing the decision‐making process (Gottfreðsdóttir and Árnason 2011). Our framework is designed to prioritize the quality of the decision‐making process rather than promoting specific outcomes (shared decision‐making). This process‐oriented approach supports diverse interpretations of seriousness, recognizing that such appraisals—and the resulting decisions—may evolve over time. In this way, the framework ensures that all relevant considerations are addressed, enabling couples to make decisions that reflect their personal values and priorities (i.e., what is most important to them).

Although elements of the framework reflect existing genetic counseling approaches and practices (e.g., the Reciprocal Engagement Model, the teaching model, the counseling model, the model of epistemic empathy) (Betzler and Roberts 2025; Veach et al. 2007), it represents the first synthesis of insights drawn from research, expert opinion, and lived experience. This integration builds on existing professional guidance and offers a holistic framing for discussing seriousness that recognizes its role in daily experiences, detached from clinical diagnosis. In doing so, the framework provides an additional, practical tool that genetic counselors can draw upon in their work and incorporate into their existing set of resources. Its structured, evidence‐informed approach to navigating conversations about seriousness serves to complement and enhance—rather than replace or duplicate—existing counseling approaches. The framework can help facilitate more organized, clear, and collaborative discussions, and further contribute to greater clarity, consistency, and depth in these inherently complex, patient‐centered conversations. As such, the framework is likely to be useful in practice for non‐genetics healthcare professionals, whereas also serving as a valuable resource for genetics health professionals in structuring their conversations around the seriousness of a genetic condition. The framework could also be used in interprofessional, cross‐disciplinary conversations between prenatal care professionals, as it establishes a shared structure and provides a common language for discussing seriousness that reduces miscommunication and facilitates consistent messaging across prenatal care teams.

### 3.1 Applying Our Framework to Case #1

BOX 1 **Rosalie and Archer Receiving Information About Down Syndrome (Trisomy 21).**

**Table:** 

Rosalie and Archer are parents to two children and are expecting their third child. Rosalie is in her late 30s. She has decided to undergo non‐invasive prenatal testing (NIPT)—also known as cell‐free prenatal DNA screening—for aneuploidies, which was offered as part of routine prenatal care. The screening indicated a screen positive result (i.e., an increased chance of Down syndrome), with the recommendation that any decision about the pregnancy should only be made following additional diagnostic testing. She met with her prenatal care professional (obstetrician) to discuss what this means and explore options for next steps. Following this discussion, Rosalie decided to have diagnostic testing (amniocentesis), and the results confirmed that the fetus has Down syndrome. Down syndrome is a chromosomal condition caused by the presence of an extra chromosome 21. The presence of this additional chromosome affects physical and cognitive development on a broad spectrum, so there is no ability to predict the impact for any particular child. A fetal echocardiogram at 20 weeks of gestation (as recommended for fetuses with Down syndrome) shows that the fetus does not have a congenital heart anomaly, the most significant physical condition associated with Down syndrome. Rosalie was referred to a genetic counselor to discuss these results and their implications, as well as available reproductive options (i.e., termination or continuation of the pregnancy). The discussion with the genetic counselor would consider the seriousness of Down syndrome from her own and Archer's perspectives.
Rosalie and Archer are both employed full‐time. They live in a metropolitan area with good access to both healthcare and educational facilities. The family is of median socioeconomic status and have strong religious beliefs. They also live close to both immediate and extended family. Rosalie and Archer have some second‐hand or vicarious knowledge of the day‐to‐day experience of raising a child with Down syndrome, as Archer's boss has a daughter with this condition. Nonetheless, they are concerned about the potential implications of parenting a child with Down syndrome and the impact this may have on all their lives. Considering the seriousness of Down syndrome will help the couple to make an informed decision about how they proceed.

#### 3.1.1 How Could Our Framework Assist With Prenatal Decision‐Making and the Expectant Parents' Appraisal of Seriousness in This Context?

Our framework can be used throughout Rosalie's pregnancy, in recognition of the fact that expectant parents need different levels of support at different time points, and that their understandings of seriousness will likely change over time.

Typically, at the initial appointment following NIPT, the increased chance or probability for Down syndrome will be the focus of discussion between Rosalie and her prenatal care professional (i.e., her obstetrician). Here, the discussion is likely to focus on general information from each core dimension and their potential implications in the expectant parents' prenatal decision‐making process. The discussion may canvas basic information about the most common health aspects related to Down syndrome (e.g., developmental delay, intellectual disability, probability of heart defect), the available healthcare, the available support (practical, financial, social), and the couple's previous experiences with Down syndrome, as the expectant parents begin to define seriousness. Given the screening results, the discussion will likely also include options for subsequent diagnostic testing, as the couple had expressed interest in receiving more certain information. Their preferences, values, and circumstances can further inform their prenatal care professional about the level of detail desired for each item and which factors weigh most heavily in their decision‐making.

In the meeting following diagnostic testing for Down syndrome, the discussion between Rosalie and her prenatal care professionals (i.e., her obstetrician and a genetic counselor) can delve deeper into each core dimension, based on Rosalie's support needs. The conversation around medical factors will be more focused on how to look for and manage specific findings, such as the need for a fetal echocardiogram and third trimester ultrasound, and other required medical follow‐ups for the child (e.g., thyroid function, spine development, hearing loss). Information about treatment will address available care for specific findings. Available support and resources in the community and their locality (e.g., schools with appropriate support), as well as support from government programs will be explained as it pertains to their situation. The experiences of children with Down syndrome and their families will also be discussed in greater detail, with accurate and up‐to‐date knowledge of Down syndrome. Information about local support or advocacy groups should also be offered. See Table 2 for an example of considerations that could be discussed with Rosalie and Archer to help them consider the seriousness of Down syndrome. If Rosalie indicates that she does not want or need additional support regarding any of the core dimensions to reach a decision, her prenatal care professionals should be sensitive to her needs. The prenatal care professionals can make note of her choice and should more support be required at any point in time, this can be revisited.

**TABLE 2:** Example of considerations that may be relevant to discuss with Rosalie and Archer as they relate to the core dimensions of seriousness in their specific circumstances.

Core dimensions	Considerations
Medical factors	Rosalie and Archer are informed that their fetus does not have a congenital heart anomaly based on fetal echocardiogram. They understand the need for a 3rd trimester ultrasound to look for duodenal atresia (an intestinal anomaly that requires surgery in the newborn period) They are comfortable with the fact that their baby may require a longer hospitalization (1–2 weeks) than other babies born at term They understand the health aspects of Down syndrome (hypotonia, delayed milestones, mild to moderate intellectual disability), including the greater probability of developing hearing loss and certain other health conditions, such as thyroid disease, leukemia, or Alzheimer's disease. They consider this manageable and are comfortable with the fact that their baby will benefit from regular follow‐ups
Access/availability of treatment	Rosalie and Archer understand that there is no specific treatment for Down syndrome They recognize that they live in an area where they have a choice of prenatal care professionals, and although there is variability in their availability, their future child would be able to have access to healthcare (medical follow‐ups, surgical care, medications, medical devices) and necessary supportive services (physical therapy, occupational therapy, and speech therapy) in a relatively timely manner Due to their socioeconomic status and stable financial situation, their out‐of‐pocket healthcare costs are comparably low and manageable
Access/availability of support and resources	Rosalie and Archer are well‐supported by family and confident that they will be able to coordinate the various healthcare services and needs their future child may require Living in a metropolitan area, they recognize that they have access to a broad range of support networks and community resources to help manage the emotional and practical aspects of raising a child with Down syndrome They also have access to early special education support (tailored educational strategies and individualized learning plans)
Individual/Familial lived experiences	Rosalie and Archer have second‐hand or vicarious experience with Down syndrome. This has helped them better understand some of the health and social aspects of living with or raising a child with this condition As they are interested in finding out more about the lived experience of individuals and their families, they have been put in contact with their local and/or national Down syndrome association Given their strong religious beliefs, they plan to seek counsel from their clergy. Their religious community discourages termination of pregnancy, influencing their ethical values and views They are confident that their family will be supportive of their child and that they have the resources to ensure that they and their child will have access to the appropriate care and supportive services given their geographic location and proximity to care

Having considered the seriousness of Down syndrome and its implications for their future child and their family, with the help of the framework and information based on their situation, Rosalie and Archer are better equipped to make an informed decision that aligns with their own values and preferences. The core dimensions will interact with expectant parents' contexts and circumstances, and as a result, similar clinical circumstances may lead to different perceptions or appraisals of a condition's seriousness. This implies that Rosalie and Archer's decision and appraisal of the seriousness of having a child with Down syndrome might change if the scenario were different across one or more of the core dimensions. The framework enables this flexibility in exploring the core dimensions to allow for more tailored information to be shared with expectant parents, including dispelling any misinformation.

### 3.2 Applying Our Framework to Case #2

BOX 2 **Harlowe and Viktor Receiving Information About Spinal Muscular Atrophy.**

**Table:** 

Harlowe and Viktor are a couple aged in their late 20s. Harlowe is 13 weeks pregnant with their first child. Given that Harlowe knows her aunt had a child with spinal muscular atrophy (SMA) who died before Harlowe was born, the couple decide to undergo genetic carrier screening to determine whether they are carriers for SMA. The results indicate that both are carriers of the common deletion in the SMN1 gene, which means they have a 25% chance (1 in 4) of having a child with SMA. SMA is a genetic condition that affects motor neurons and leads to progressive muscle weakness and atrophy. Although up to 98% of cases of SMA are due to the same mutation (common exon 7 deletion of the SMN1 gene), severity varies. The number of copies of the SMN2 gene influences the severity of this condition, but the correlation is not absolute. Almost all patients with one copy of SMN2 present with Type I (most severe form with earliest onset), whereas almost all patients with three copies or more present with Type II or Type III (Calucho et al. 2018). Apart from knowing the condition is in Harlowe's family, the couple is unfamiliar with SMA and are anxious about their carrier screening results. They turn to their prenatal care professional (general practitioner) for guidance. They are ambivalent about termination of pregnancy, but open to prenatal testing of the fetus to gain a better understanding of the situation. Their prenatal care professional refers them to a genetics clinic (genetic counselor) to discuss their options, including invasive diagnostic testing or testing of the newborn. After discussion, they opt for prenatal diagnostic testing by amniocentesis, to identify if the fetus has SMA so they can then make a decision about the pregnancy. The results of the amniocentesis indicate that the fetus is homozygous for the SMN1 exon 7 deletion and has one copy of the SMN2 gene, which means that their future child will almost certainly develop SMA Type I. However, the exact trajectory for the condition cannot be confirmed in advance. Given Harlowe and Viktor's desire for more information to ensure that they are as informed as possible about the realities of raising a child with the condition, they are seen again at the genetics clinic and meet with a pediatric neurologist to discuss the options for their baby's care once born. Harlowe and Viktor recently moved to a new country and live in a one‐storey home in a rural region with limited healthcare resources. They have concerns about public funding and reimbursement for SMA therapies in their country. Harlowe is the family's primary source of income. They have no family support nearby. They are concerned about the results and want to understand their options and the potential impact of a diagnosis of SMA on their lives and that of their future child, so that they feel better prepared and equipped to make a decision.

#### 3.2.1 How Could Our Framework Assist With Prenatal Decision‐Making and the Expectant Parents' Appraisal of Seriousness in This Context?

As with Case #1, our framework can assist in appraising seriousness at various stages during Harlowe's pregnancy. It is relevant both following the identification of Harlowe and Viktor's carrier status and the results of the diagnostic test.

In the initial meeting following the results indicating they are both carriers, the discussion between Harlowe, Viktor, and their prenatal care professionals (general practitioner and genetic counselor) will focus on exploring the couple's values and beliefs regarding prenatal testing, fetal surveillance, and newborn testing. The discussion can also include general information covering all four core dimensions (i.e., medical factors about SMA Type I, available therapies and supportive care, available support and resources, and individual/familial experiences) as the couple begin to define and delineate the seriousness of SMA from their perspective.

In the meeting following the results of the amniocentesis and diagnosis of SMA (most likely Type I), the discussion between the expectant parents and their prenatal care professionals (genetic counselor and pediatric neurologist) will cover each of the core dimensions of the framework in more depth and center around care options available to their child after birth. The discussion around medical factors will be more focused on the potential clinical manifestations, natural history of the disease, and disease progression for SMA Type I. Available therapies and supportive care, along with out‐of‐pocket costs, will be explored with attention to Harlowe and Viktor's particular circumstances. The discussion around resources may focus on available support for families (online forums or advocacy groups). Limits to other forms of financial assistance, counseling, respite care, and possible alternatives can also be explored with the couple based on their situation and values. See Table 3 for an example of considerations that could be discussed with Harlowe and Viktor to help them appraise the seriousness of SMA and different care options available to their child after birth. This approach will allow for a broader exploration of Harlowe and Viktor's understanding of seriousness and what the diagnosis may imply for them, the provision of more targeted information, and the impact of their decision to continue or terminate the pregnancy for their family.

**TABLE 3:** Example of considerations that may be relevant to discuss with Harlowe and Viktor as they relate to the core dimensions of seriousness in their specific circumstances.

Core dimensions	Considerations
Medical factors	Harlowe and Viktor understand that their baby will develop SMA, most likely Type I They understand the rapid progression of the condition (developmental motor regression), including early onset (< 6 months) and early mortality, if untreated
Access/availability of treatment	Harlowe and Viktor understand there is no cure for SMA They understand the options available to them for the care of their child after birth are to treat them with a targeted therapy such as Evrysdi (risdiplam); Spinraza (nusinersen); Zolgensma (onasemnogene abeparvovec) or supportive care (nutritional supportive care, non‐invasive or invasive respiratory support, antibiotics for recurrent infections, and rehabilitative support) (Mercuri et al. 2022, 2020; Gowda et al. 2023; Crisafulli et al. 2023). Harlowe and Victor understand the importance of early intervention. They have also discussed and considered the methods of administering each therapy: Evrysdi is taken orally once daily (lifelong), Spinraza requires regular administration via spinal injections (lifelong), and Zolgensma is a one‐time intravenous injection (gene therapy) All three targeted therapies are available in their country on a case‐by‐case assessment and would incur significant out‐of‐pocket costs for them (financial burden), if not reimbursed. However, their future child could be a good candidate for access to Zolgensma They also considered that Spinraza requires procedure visits every 4 months once the initial dosing schedule is complete, and that this could be physically burdensome for their child They understand that the targeted therapies could potentially increase how long their child lives, but that long‐term data is still lacking
Access/availability of support and resources	Harlowe and Viktor recognize that because they live in a rural region, access to healthcare resources, counseling, and respite care to help manage the emotional and practical challenges of raising a child with SMA is limited They note that access to either targeted therapies or even supportive care will be logistically challenging as the closest prenatal care professionals with experience with SMA are located more than 4 h away As Harlowe is the family's primary source of income, she is concerned about the practical and financial burden of taking time off work to coordinate and bring their child to frequent and regular appointments Although online resources exist to support families emotionally and provide practical advice, the couple feel that without family nearby to provide additional support and limited access to financial assistance, the impact of caring for a child with SMA Type I is significant
Individual/Familial lived experiences	Harlowe and Viktor do not have any personal experience with SMA, despite Harlowe's family history. They feel overwhelmed and anxious about the possibility of having a child with SMA Type I They acknowledge that ongoing treatment, physiotherapy, clinical surveillance, and regular monitoring will have a significant impact on their daily lives As recent immigrants, access to a trained health translator would be helpful to ensure the support is being provided in a manner that ensures clear and culturally sensitive communication Their recent move has the couple still adjusting to new systems and norms Being able to contact an advocacy or support group and receive experiential knowledge from individuals with recent experience of an SMA Type I diagnosis and their families appeased some of their concerns and made the diagnosis feel more manageable for them They recognize that a minority of children with SMA Type I may not respond to treatment and may continue to experience significant motor impairments (Goedeker et al. 2024). In the event that their child develops mobility limitations despite treatment, modifications may also be required to their home environment to accommodate their future child's evolving needs (e.g., wheelchair accessible)

Having considered the seriousness of SMA Type I and its implications for their future child and their family, based on their situation, Harlowe and Viktor are better equipped with knowledge and resources to make an informed decision that aligns with their own values and preferences. Lived experience (experiential knowledge) shapes a person's beliefs, perceptions, and decision‐making (Swainson et al. 2024; Montgomery and Thayer 2020). Such information is valuable in helping a person or couple understand what it might be like to have children with a genetic condition, but consideration of seriousness may vary with each contextualized application of the core dimensions. Therefore, even if Harlowe and Viktor had second‐hand or vicarious knowledge of SMA through Harlowe's aunt, the discussion around SMA is very different today than it would have been for Harlowe's aunt, as the reality of life with SMA has significantly changed (i.e., longer survival and better symptomatic treatment).

### 3.3 Applying Our Framework to Case #3

BOX 3 **Priya and Amir Receiving Information About a Variant of Uncertain Significance.**

**Table:** 

Priya, a 29‐year‐old woman in her first pregnancy, is referred to a tertiary fetal medicine unit, by her prenatal care professional (obstetrician), at 21 weeks gestation after her routine fetal anatomy scan reveals mild bilateral ventriculomegaly (slightly enlarged brain ventricles), mild limb contractures, and normal cardiac and abdominal anatomy. Priya and her partner, Amir, are healthy, with no known family history of genetic conditions. Given these findings, a fetal MRI and amniocentesis, for chromosomal microarray and fetal exome sequencing, are offered to further investigate the ultrasound findings. The possibility that the tests could reveal a variant of uncertain significance (VUS) was mentioned to Priya and Amir. The couple agrees to proceed and to receive information about a VUS if one is found. The fetal MRI confirms mild ventriculomegaly and suggests mild cerebellar hypoplasia. A VUS is identified in the *KIF1A* gene, associated with a spectrum of neurodevelopmental conditions. Priya and Amir are referred to the prenatal genetics clinic to discuss these results and consider the nature of the VUS (and its implications) from their perspective, including possible next steps. At 28 weeks of gestation, a follow‐up ultrasound reveals progressive ventriculomegaly, arthrogryposis of the lower limbs, and polyhydramnios. These findings are all highly suggestive of significant neurological involvement (neurodevelopmental impairment) in the fetus. Priya and Amir meet with a multidisciplinary team including a medical geneticist, fetal medicine specialist, genetic counselor, and perinatal psychologist to discuss the results and what options are available to them both prenatally and postnatally. They are particularly concerned about the potential seriousness of the condition and its impact on their family. Priya and Amir are both employed full‐time. The family is upper socioeconomic status. The couple have a multicultural background. Priya's parents and siblings all live nearby and regularly see each other. They live in a metropolitan area within proximity to high quality hospitals and genetic counseling services. The couple struggled with infertility for years and became pregnant via in vitro fertilization (IVF). As such, they are reluctant to consider terminating the pregnancy.

#### 3.3.1 How Could Our Framework Assist With Prenatal Decision‐Making and the Expectant Parents' Appraisal of Seriousness in This Context?

As with Cases #1 and #2, our framework can assist in appraising seriousness throughout Priya's pregnancy. It is relevant following the results of the routine fetal anatomy scan, the results of the fetal MRI and amniocentesis, and the results of the follow‐up ultrasound.

In the initial meeting following the routine fetal anatomy scan, Priya, Amir, and their prenatal care professional (obstetrician) will discuss the findings as they explore the couple's values, beliefs, and circumstances. The four core dimensions of the framework can provide a structure as the couple begins to explore the potential seriousness of the initial findings based on general information about the clinical implications of the potential diagnosis, the need for more specialized care (including available support and resources), recommended next steps to further clarify the findings and assess possible genetic causes, including the potential for uncertain outcomes. These will be discussed in light of the couple's current circumstances and their IVF journey.

Following the results of the fetal MRI and amniocentesis (for chromosomal microarray and fetal exome sequencing), the discussion between the expectant parents and their prenatal care professional (genetic counselor) will explore each of the core dimensions further based on the new information that has become available. The conversation around medical factors will focus on the potential clinical manifestations and impacts of mild ventriculomegaly and mild cerebellar hypoplasia. The discussion will also include information related to the identified VUS in the KIF1A gene, as it is linked to a spectrum of neurodevelopmental conditions. This includes exploring the implications for fetal development (i.e., what is known, what is unclear, and what might change over time). The possible prognoses and need for follow‐up testing will also be discussed. Psycho‐social resources available to the couple to help them cope with ambiguous results and uncertainty will be explained.

After the follow‐up ultrasound, the discussion between Priya, Amir, and the multidisciplinary team of prenatal care professionals (geneticist, fetal medicine specialist, genetic counselor, and perinatal psychologist) will explore each of the core dimensions in more depth. The discussion around medical factors will focus on the clinical manifestations of progressive ventriculomegaly, arthrogryposis of the lower limbs, and polyhydramnios. They will also discuss the possibility that the classification of the VUS may change as new knowledge is gained, but for now the significance of the VUS remains uncertain. The availability of treatment options and supportive care (prenatal and postnatal), as well as pregnancy management (early interventions, birth planning, and long‐term considerations) will be explored for each of the findings with particular attention to the expectant parents' circumstances, values, and preferences. Access and availability to support and resources will also be considered, including psychological and emotional support. The expectant parents' experience and familiarity with the information, their familial relationships, and circumstances must also be considered. See Table 4 for an example of considerations that could be discussed with Priya and Amir to help them appraise the seriousness of progressive ventriculomegaly, arthrogryposis of the lower limbs, polyhydramnios, and the VUS, including the uncertainty and possible options that are available to them and their child after birth. This helps the team of prenatal care professionals to explore what matters most to Priya and Amir—how they define quality of life, caregiving capacity, cultural or religious beliefs, level of preparedness—and tailor the discussions around these relevant elements.

**TABLE 4:** Example of considerations that may be relevant to discuss with Priya and Amir as they relate to the core dimensions of seriousness in their specific circumstances.

Core dimensions	Considerations
Medical factors	Priya and Amir are informed that their fetus has some atypical findings on imaging—progressive ventriculomegaly (evolving neurological involvement, increasing the concern for long‐term cognitive or motor impairments); cerebellar hypoplasia and arthrogryposis (significant motor and coordination challenges, possibly lifelong and disabling for their future child); polyhydramnios (often associated with neurological involvement and compromised swallowing function, which can add complexity to perinatal and neonatal care needs for their future child) They recognize that the VUS does not currently provide prognostic value regarding the child's future outcomes. Any potential prognosis is instead informed by the ultrasound findings, which suggest an underlying syndrome with possible neurodevelopmental implications They understand that the classification of the VUS may change as new information becomes available. Should the variant be reclassified as pathogenic or likely pathogenic, it may then offer more meaningful guidance for prognosis assessment If they choose to continue the pregnancy, they are aware that postnatal follow‐up would involve early intervention and developmental monitoring based on the signs of neurological involvement on the ultrasound
Access/availability of treatment	The couple understand that there is no specific treatment for KIF1A‐related conditions They live near major hospitals and have access to fetal medicine, neurology, neonatal intensive care, and early intervention services (proximity to tertiary care) As financially stable professionals, they are able to cover out‐of‐pocket healthcare costs The couple have experienced infertility and became pregnant via IVF, after several years of trying. As a result, they are reticent to terminate the pregnancy and are keen to explore medical follow‐up once the baby is born
Access/availability of support and resources	They recognize that they have access to a multidisciplinary care team that enables nuanced understanding and emotional support, both prenatally and postnatally They are reassured by the balanced options that have been presented to them: a multidisciplinary birth plan with neonatology, neurology, and social work in place, depending on the newborn's condition. The team also discussed the potential for the child to participate in variant tracking studies once born With family nearby, they are confident that they can benefit from caregiving, emotional support, and decision‐making collaboration
Individual/Familial lived experiences	After experiencing infertility, this pregnancy has particular significance for Priya and Amir (noted above) They recognize that there are practical implications of caring for a child with potentially complex needs—affecting career paths, daily routines, and long‐term planning Coming from distinct cultural backgrounds, their families have differing views on disability and medical intervention, which adds layers to their deliberation Priya struggles with ambiguity and expresses fear of stigma and guilt. She is grateful that emotional support resources, including perinatal psychologists, were offered Amir feels overwhelmed by the complexity and fears making the “wrong” choice They are comfortable navigating complex care systems Wanting to take a proactive approach, Priya connected with other expectant parents through online support groups

In light of the evolving clinical findings—progressive ventriculomegaly, lower limb arthrogryposis, polyhydramnios, Priya and Amir, in consultation with their prenatal care team and guided by the framework and information tailored to their context, are now well‐positioned to make an informed decision that reflects their personal values, priorities, and family aspirations. In Priya and Amir's case, the interplay between a medically complex presentation, including uncertainty of outcomes, and their current circumstances and IVF journey makes the situation all the more layered. The couple understands that the clinical findings cannot be definitively attributed to the presence of the VUS and that, if they are related to the VUS, the seriousness of the genetic condition related to the VUS is difficult to predict and can evolve over time. This evolving clinical picture requires sensitive, clear, and collaborative communication with and between the prenatal care professionals to ensure that the couple's circumstances are being considered and that their concerns or struggles related to the uncertainty are being addressed. The framework enables prenatal care professionals to balance clinical information with contextual factors that are directly relevant to Priya and Amir's reproductive decision‐making to ensure they feel supported throughout.

In discussing these cases, our aim is not to present particular outcomes and their links to perceptions of seriousness. Rather, we seek to illustrate how our framework offers prenatal care professionals a structured approach for exploring the elements most relevant to each couple's understanding of seriousness, whereas also creating space for expectant parents to reflect on their own meanings, values, circumstances, and preferences. The framework helps prenatal care professionals counsel couples and individuals experiencing prenatal diagnoses by guiding the exploration of medical implications (including progression of the condition), treatment availability and associated burdens, support systems, and personal values. It allows prenatal care professionals and expectant parents to explore care options and the implication of the diagnosis and their decision in their circumstances, covering elements in all four core dimensions (as needed or relevant). These should be framed within considerations of the procedural elements, namely patient/family‐centered impacts and shared decision‐making. The framework, therefore, may be useful at several points in practice: before discussions or clinical encounters, as a preparatory and training tool; during discussions, as a flexible guide to support responsive and patient‐centered engagement; and following encounters, as a means of reflecting on how considerations of seriousness were raised, interpreted, and communicated. Its value, in our view, lies in supporting thoughtful, context‐sensitive practice rather than procedural completeness.

For genetic counselors and other genetics professionals, the framework serves as a practical tool in situations where concerns about seriousness are central for expectant parents and where an explicit, structured appraisal of seriousness would be beneficial. It can also further assist or guide non‐specialist healthcare professionals, who may be less accustomed to having such conversations. The framework helps to ensure that prenatal care professionals address not only the medical factors but all four core dimensions, thereby offering better support to expectant parents by providing relevant information as they begin their reflections around seriousness in prenatal decision‐making. This aims to help patients contextualize seriousness and make decisions aligned with their situation. It is also useful in cases where there is uncertainty, helping expectant parents navigate unknowns by addressing evolving genomic information, uncertainty in prognosis, and psychosocial factors to support informed decision‐making despite incomplete information. In this way, the framework remains adaptable to context and highly responsive to the unique circumstances of expectant parents. As the central participants in these conversations, expectant parents guide the exploration of topics covered based on what is most relevant and meaningful to them. At the same time, prenatal care professionals' affect, communication style, and personal appraisals of seriousness may shape both how information is conveyed and how it is interpreted by expectant parents. We acknowledge that clinical neutrality is not always fully attainable or apparent in these interactions, that non‐directiveness can come at a cost, and that reflexive awareness of one's own assumptions about seriousness is an essential first step in facilitating thoughtful discussion while minimizing unintended influence on decision‐making. Accordingly, prenatal care professionals should be equipped to recognize and mitigate potential biases, as well as public or personal value judgments, when supporting individuals and families living with a range of genetic conditions (Jamal et al. 2020). We also recognize that additional circumstances and factors—such as the timing of conversations about seriousness and the various prenatal care professionals involved at different stages of pregnancy—are important and warrant further exploration. How considerations of seriousness are addressed may vary across different points in the prenatal testing process and according to the prenatal care professionals involved, each bringing distinct roles, expertise, and perspectives on seriousness.

In practice, conversations about seriousness are shaped by a range of emotional, relational, and situational factors that influence how information is understood and acted upon. This applies both to expectant parents, who may be navigating these discussions amidst uncertainty, distress, hope, or conflicting values, and to prenatal care professionals, whose own affective responses and interpretative assumptions may also shape the clinical encounter. Accordingly, whereas the framework is designed to support structured reflection on appraisals of seriousness, its application should remain attentive to capture the affective complexity of the clinical situation. The framework should therefore be understood as one component of a broader, relational, and emotionally responsive approach to prenatal care.

## 4 Conclusion

In this article, we have presented how our previously published framework for weighing and evaluating the seriousness of a genetic condition can be used when providing prenatal care. The framework addresses a key conceptual challenge for determinations of seriousness that whilst seriousness might be the property of a condition or of an instance of it in a particular person (Dive et al. 2023), it is nevertheless experienced in a highly contextual way, inflected by the individual or couple's familial, socio‐cultural, and environmental circumstances, as well as lived experiences (e.g., infertility and experiences of health and illness). The detection and diagnosis of a genetic condition is the gateway into these conversations, but will always be limited in terms of how far it can account for the way the condition will be lived, and its meaning to the family. Our framework addresses the challenge that clinical interpretations of seriousness often prioritize clinical information, which can be at the expense of other contextual and personal factors critical to expectant parents' decisions. It aims to broaden discussions to include these dimensions for more meaningful counseling.

Although genetic counselors are well positioned to provide counseling related to prenatal diagnoses, they increasingly practice within interdisciplinary and resource‐constrained systems where counseling may be fragmented, time‐limited, or partially delegated. Presenting the framework in a manner accessible to non‐specialists reflects the realities of contemporary practice and offers a shared language to support interdisciplinary discussions about seriousness. It also underscores process‐oriented skills aligned with core genetic counseling competencies and demonstrates how culturally responsive, patient‐centered care can be delivered consistently across diverse clinical settings. Thus, rather than introducing skills beyond the existing scope of practice, the framework serves as a reflective tool for both genetic counselors and non‐genetics healthcare professionals to support ethically robust practice, education, and supervision.

The framework, through its core dimensions, facilitates shared meaning‐making and tailored information exchange between expectant parents and prenatal care professionals. It supports iterative, flexible conversations that reflect the variability in how seriousness is perceived by different individuals or couples over time and circumstances, promoting autonomy and deliberation. Although the framework was developed around prenatal and reproductive decision‐making for heritable or congenital genetic and chromosomal conditions, it was designed to be transferable to other scenarios and contexts where guided reflections on seriousness are already taking place, for example in health policy and/or resource allocation decisions (UK National Screening Committee 2022; Stenmarck et al. 2023; Kleiderman et al. 2026). The framework has already been successfully employed in an exploration of preimplantation genetic testing decisions for conditions such as hereditary cancer and retinoblastoma in Japan (Takahashi et al. 2025), and we encourage further consideration of the relevance of our framework and its applications beyond genomic and reproductive medicine, including its use within contrasting cultures, healthcare systems, and environmental conditions.

## Author Contributions

**Erika Kleiderman:** conceptualization, methodology, writing – original draft, writing – review and editing. **Anne‐Marie Laberge:** conceptualization, methodology, writing – review and editing. **Ainsley J. Newson:** conceptualization, methodology, writing – review and editing. **Lucinda Freeman:** conceptualization, methodology, writing – review and editing. **Vardit Ravitsky:** conceptualization, methodology, supervision, writing – review and editing. **Felicity Boardman:** conceptualization, methodology, writing – review and editing. All authors read and approved the final manuscript. This work was conducted in the context of E.K.'s doctoral dissertation in fulfillment of a degree requirement.

## Funding

The authors acknowledge the funding and support provided by the Brocher Foundation. Additional sources of funding include from Genome Canada, Génome Québec, and the Canadian Institutes of Health Research (CIHR)—PEGASUS‐2 (VR and AML); the Medical Research Future Fund (Australia), Genomics Health Futures Mission (GHFM), Grant GHFM73390 (MRFF‐G‐MM) (AJN and LF); an Australian Research Council Discovery Project (grant DP200100006) (AJN); and the Wellcome Trust Investigator Award (2033884/Z/16/Z) (FB). AML receives salary support from the Fonds de recherche du Québec Clinical Research Scholar—Senior Grant.

## Ethics Statement

The authors have nothing to report.

## Consent

The authors have nothing to report.

## Conflicts of Interest

The authors declare no conflicts of interest.

## Data Availability

The authors have nothing to report.

## Endnotes

^1^
Also known as cell‐free fetal DNA testing.

^2^
The concepts of “serious” and “severe” are used synonymously within this article. However, we recognize that “serious” may be more appropriate for capturing the experiential and contextual aspects of a condition, whereas “severe” typically has a more clinical or biomedical connotation.
